# Impairment of the CD8+ T cell response in lungs following infection with human respiratory syncytial virus is specific to the anatomical site rather than the virus, antigen, or route of infection

**DOI:** 10.1186/1743-422X-5-105

**Published:** 2008-09-24

**Authors:** Joshua M DiNapoli, Brian R Murphy, Peter L Collins, Alexander Bukreyev

**Affiliations:** 1 Laboratory of Infectious Diseases, National Institute of Allergy and Infectious Diseases, National Institutes of Health, 50 South Drive, Room 6505, Bethesda, Maryland, 20892, USA

## Abstract

**Background:**

A subset of the virus-specific CD8+ cytotoxic T lymphocytes (CTL) isolated from the lungs of mice infected with human respiratory syncytial virus (RSV) is impaired in the ability to secrete interferon γ (IFNγ), a measure of functionality. It was suggested that the impairment specifically suppressed the host cellular immune response, a finding that could help explain the ability of RSV to re-infect throughout life.

**Results:**

To determine whether this effect is dependent on the virus, the route of infection, or the type of infection (respiratory, disseminated, or localized dermal), we compared the CTL responses in mice following intranasal (IN) infection with RSV or influenza virus or IN or intradermal (ID) infection with vaccinia virus expressing an RSV CTL antigen. The impairment was observed in the lungs after IN infection with RSV, influenza or vaccinia virus, and after a localized ID infection with vaccinia virus. In contrast, we observed a much higher percentage of IFNγ secreting CD8+ lymphocytes in the spleens of infected mice in every case.

**Conclusion:**

The decreased functionality of CD8+ CTL is specific to the lungs and is not dependent on the specific virus, viral antigen, or route of infection.

## Background

Recently, it was shown that infection of mice with RSV results in the induction of CD8+ CTL in lungs that are characterized by a low percentage of cells secreting IFNγ, which is a direct measure of their cytolytic activity [1]. It was also demonstrated that the percentage of RSV-specific CTL secreting IFNγ in the lungs quickly decreased within a few weeks, consistent with previous studies that showed a rapid reduction in RSV-specific CD8+ cells in the lungs and in the protective effect they conferred against re-infection [2]. The impairment in the expression of IFNγ suggested that RSV specifically suppresses the host cellular immune response at both the effector and memory phases, a finding that could help explain the propensity for RSV to re-infect throughout life. However, more recent studies have called into question the finding that RSV specifically mediates suppression of lymphocytes in lungs, as a similar effect was observed following infection with simian virus 5 (SV5) [3], influenza virus [4], and pneumonia virus of mice, a relative of RSV [5].

## Results and discussion

We attempted to determine (i) whether the impairment of IFNγ production by CD8+ CTL in the lungs depends on the viral context (i.e., expression of antigen by RSV versus a heterologous live viral vector); (ii) whether the impairment is antigen-specific, (iii) whether a similar impairment is observed following primary versus secondary infection; (iv) whether the impairment is observed after a non-respiratory infection, and (v) whether there is a difference in the percentage of virus-specific IFNγ + CD8+ T cells in the lungs versus the spleen after respiratory and non-respiratory infections. We used two respiratory viruses, RSV (strain A2) and influenza virus A/Puerto Rico/8/34 (H1N1), which were administered IN, and a non-respiratory virus, a recombinant Western Reserve (WR) strain of vaccinia virus (VV) expressing the RSV M2-1 protein (VV-M2), which was administered either by the IN or ID route. IN inoculation of mice with the WR strain of VV has been shown to cause respiratory tract infection followed by dissemination of the virus to various visceral organs and the brain [6,7]. In contrast, ID inoculation with the virus has been shown to result in a highly localized infection without spread of the virus to internal organs [8]. In addition, following tail skin scarification of mice with the same virus, no viral DNA was detected in various lymph nodes distant from the site of initial infection by a highly sensitive quantitative PCR [9]. The VV-M2 virus used in the present study contains a disrupted thymidine kinase gene due to the M2 insert [10]. This disruption has been shown to result in attenuation compared to its strain WR parent, yet the virus still causes disseminated infection following IN inoculation [6,11,12].

In the present study, we first compared pulmonary replication of the VV-M2 virus after infection by either the IN or ID route. Groups of BALB/c mice were infected with 10^5 ^PFU of VV-M2 by either route and were sacrificed on days 2, 4 and 6 post-infection (two and four animals per day for IN and ID infection, respectively). The lungs were isolated from each animal, and viral titers in the tissue were determined by plaque titration of lung homogenates. In animals infected by the IN route, the following titers (log_10 _PFU per g of lung tissue) were detected in the two animals euthanized on each day: day 2, 2.9 and <2.0; day 4, 5.1 and 5.0; and day 6, 2.3 and <2.0. In contrast, no virus was detected in the lungs of any of the four ID-infected mice on any day.

We next used BALB/c mice to monitor CD8+ CTL responses to the M2-1 protein expressed by RSV versus VV-M2 using a peptide, SYIGSINNI, from the M2-1 protein (amino acids 82 to 90) that is the immunodominant CTL epitope in the H-2Kd background [10]. Thus, the same RSV epitope was presented in the context of two distinct viruses (RSV versus vaccinia virus). The CD8+ CTL response to influenza virus was monitored using a peptide, TYQRTRALV, from the nucleoprotein NP (amino acids 147–155) that is the immunodominant CTL epitope in the H-2Kd background [13]. CD8+ CTL specific to the RSV M2-1 or influenza virus NP peptide epitope were quantified by staining with phycoerythrin-conjugated MHC class I H-2K^d ^tetramer (RSV) or pentamer (influenza virus) complexes loaded with the respective M2-1 or NP peptide. In addition, intracellular IFNγ staining was performed following in vitro stimulation with the respective peptides.

To compare the primary CD8+ CTL responses to various viruses, mice were infected with 10^5 ^PFU of RSV administered by the IN route, or 10^4 ^50% tissue culture infectious doses of influenza virus administered by the IN route, or 10^5 ^PFU of VV-M2 administered by the IN or ID route (Table 1). On days 8 and 28 after infection, total pulmonary mononuclear cells (PMC) and total spleen mononuclear cells (SMC) were isolated [14] and were analyzed to quantify the number of CD8+ CTL that were positive for binding to the MHC class I tetramer (RSV M2-1) or pentamer (influenza virus NP) mentioned above, or for intracellular IFNγ staining following in vitro stimulation with the M2-1 or NP peptide [15]. This experimental design allowed us to analyze the dependency of the CD8+ CTL response in the lung and the spleen on the viral context (i.e. M2 expressed by RSV versus that expressed by VV-M2), the viral antigen (RSV M2-1 versus influenza virus NP), and the location of infection (pulmonary versus disseminated versus localized dermal). To examine the secondary CD8+ CTL responses, groups of mice were mock-infected or infected with RSV or influenza virus by the IN route, or with VV-M2 by the IN or ID route, as above. Thirty-four days later, the animals were secondarily infected with RSV or influenza virus by the IN route, or with VV-M2 by the IN or ID route, as above (Table 1). Eight and 28 days following the second infection, lungs and spleens were isolated and CD8+ CTL analyzed.

**Table 1:** Virus-specific tetramer/pentamer+ CD8+ T cells and IFNγ + CD8+ T cells in the lungs and spleens of mice following primary and secondary infections with the indicated viruses (% of total CD8+ cells)

	Days after primary (secondary) infection	Lung		Spleen	
		
		Tet+CD8+/total CD8+, %	IFNγ+CD8+/total CD8+, %	Tet+CD8+/total CD8+, %	IFNγ+CD8+/total CD8+, %
Primary Infection^*a*^					

Mock^*b *^(N = 2)	8	2.3	0.03	0.52	0.14
RSV^*b *^(N = 5)	8	23 ± 2.2	5.8 ± 0.21	3.5 ± 0.32	3.2 ± 0.56
VV-M2 IN^*b *^(N = 5)	8	15 ± 1.4	5.2 ± 0.56	3.8 ± 0.51	2.2 ± 0.22
VV-M2 ID^*b *^(N = 5)	8	21 ± 2.1	8.9 ± 1.0	8.7 ± 0.85	8.2 ± 1.3
Mock^*c *^(N = 2)	8	1.3	0.08	0.53	0.04
Flu^*c *^(N = 5)	8	30 ± 1.9	5.6 ± 0.25	3.4 ± 0.20	1.4 ± 0.18

Mock^*b *^(N = 2)	28	0.35	0.03	0.14	0.06
RSV^*b *^(N = 5)	28	4.2 ± 0.60	0.58 ± 0.07	1.4 ± 0.22	1.5 ± 0.27
VV-M2 IN^*b *^(N = 5)	28	6.8 ± 0.76	0.87 ± 0.10	1.2 ± 0.13	1.1 ± 0.11
VV-M2 ID^*b *^(N = 5)	28	5.1 ± 0.70	1.9 ± 0.32	3.0 ± 0.50	4.2 ± 0.51
Mock^*c *^(N = 2)	28	0.46	0.01	0.30	0.07
Flu^*c *^(N = 5)	28	16 ± 2.7	1.8 ± 0.23	0.92 ± 0.14	0.53 ± 0.15

Secondary Infection^*d*^					

Mock; Mock^*b *^(N = 1)	42 (8)	0.30	0.46	0.16	0.34
Mock; RSV^*b *^(N = 5)	42 (8)	30 ± 2.7	6.6 ± 0.85	5.7 ± 0.84	5.6 ± 0.73
RSV; RSV^*b *^(N = 5)	42 (8)	53 ± 1.5	9.5 ± 0.57	3.3 ± 0.19	2.7 ± 0.46
RSV; VV-M2 IN^*b *^(N = 5)	42 (8)	46 ± 0.75	11 ± 1.1	4.2 ± 0.48	2.9 ± 081
RSV; VV-M2 ID^*b *^(N = 5)	42 (8)	22 ± 2.0	5.9 ± 0.50	14 ± 3.3	9.7 ± 1.9
Mock; Mock^*c *^(N = 1)	42 (8)	1.1	0.62	1.1	0.08
Flu; Flu^*c *^(N = 5)	42 (8)	20 ± 1.8	4.5 ± 0.16	4.5 ± 0.86	3.3 ± 1.0

Mock; Mock^*b *^(N = 1)	62 (28)	0.21	0.38	0.31	0.49
Mock; RSV^*b *^(N = 5)	62 (28)	6.0 ± 0.68	2.0 ± 0.25	1.3 ± 0.15	1.3 ± 0.20
RSV; RSV^*b *^(N = 5)	62 (28)	11 ± 2.9	1.3 ± 0.33	3.1 ± 0.62	1.6 ± 0.33
RSV; VV-M2 IN^*b *^(N = 5)	62 (28)	16 ± 1.8	3.6 ± 0.27	3.7 ± 0.28	2.6 ± 0.30
RSV; VV-M2 ID^*b *^(N = 5)	62 (28)	14 ± 2.0	6.7 ± 0.94	7.6 ± 077	9.2 ± 0.36
Mock; Mock^*c *^(N = 1)	62 (28)	1.5	0.40	2.8	0.30
Flu; Flu^*c *^(N = 5)	62 (28)	18 ± 3.8	2.6 ± 0.51	2.9 ± 0.36	1.5 ± 0.14

^*a *^On day 0, mice were infected with 10^5 ^PFU of RSV by the IN route, 10^5 ^PFU of VV-M2 by IN or ID route, or 10^4 ^PFU of influenza A virus (Flu) administered by the IN route. On days 8 and 28 post-infection, the animals were sacrificed and total PMC and splenocytes were isolated, stained for CD8 in combination with a virus-specific MHC class I tetramer/pentamer or intracellular IFNγ staining as described in the text, and analyzed by flow cytometry.

^*b *^Analyzed with the RSV-specific MHC class I tetramer.

^*c *^Analyzed with the influenza virus-specific MHC class I pentamer.

^*d *^On day 0, mice were infected with RSV or influenza virus by the IN route or were mock-infected. On day 34, the animals were secondarily infected with RSV by the IN route, VV-M2 by the IN or ID route, or influenza virus, as indicated. Viral doses are as described in footnote *a*. PMC or splenocytes were isolated on days 42 and 62 (8 and 28 days following the secondary infection), and analyzed as above.

On day 8 following the primary infection with RSV, a robust tetramer+CD8+ T cell response (23% of total CD8+ cells) was detected in the lungs (Table 1). A somewhat lower response (15%) of tetramer+CD8+ cells was detected in the lungs after IN infection with VV-M2. Interestingly, despite the lack of VV-M2 replication in the lungs after ID inoculation, a high level (21%) of tetramer+CD8+ T cells also was detected in the lungs. Similar to RSV, IN infection with influenza virus resulted in a robust influenza virus-specific CD8+ CTL response (30%) in the lungs on day 8. In the spleen, weaker responses (3.4%–3.8%) were detected following IN infection with RSV, VV-M2, or influenza virus whereas a higher response (8.7%) was detected after ID infection with VV-M2. On day 28, the percentages of virus-specific cells were reduced substantially in both the lungs and the spleen, although in the lungs, the reduction for the influenza virus-specific cells was less than for the RSV-specific cells.

**Figure 1:**
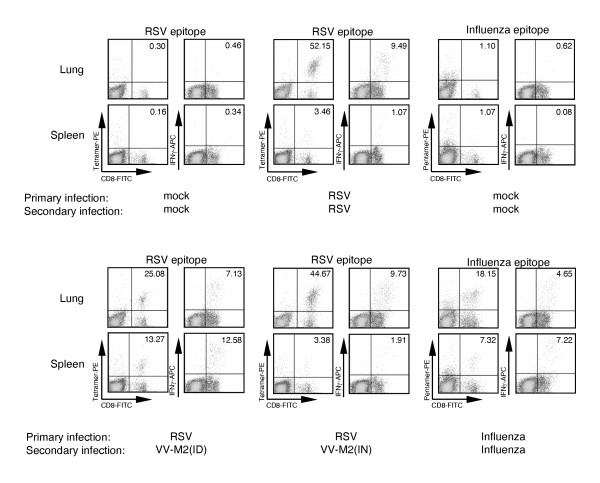
**Examples of primary data of flow cytometry analysis of tetramer/pentamer+CD8+ and IFNγ+CD8+ cells from the lungs and the spleens of individual mice.** Mice were mock-infected or infected as indicated below the plots on days 0 and 28. The animals were sacrificed 8 days later (day 36) and lungs and spleens were collected. PMC and splenocytes were isolated and stained with MHC class I tetramer or pentamer complexes specific for an RSV or influenza virus epitope or stimulated in vitro with the epitope-specific peptide, stained for intracellular IFNγ and analyzed by flow cytometry. Percentages relative to total CD8+ cells are shown for various cell populations. The data are from the experiment shown in Table 1.

After secondary IN infection of RSV-experienced mice with RSV or VV-M2, the levels of tetramer+CD8+ cells on day 8 were greater in the lungs, but not in the spleen, than after the primary infection: 53% and 46%, respectively (Table 1). After infection of RSV-experienced animals with VV-M2 by the ID route, a somewhat lower level (22%) of tetramer+CD8+ cells was detected in the lungs on day 8, which was essentially the same (21%) as after infection of RSV-naive animals. As had been observed following primary infection with VV-M2 by the ID route, there was a high percentage (14%) of positive cells in the spleen. The secondary infection of influenza-experienced animals with influenza virus resulted in a level (20%) of pentamer+CD8+ T cells in the lungs on day 8 that was not significantly increased compared to the level (16%) observed on day 28 following a primary infection. Examples of primary flow cytometry data for individual animals following secondary infection are shown in Figure 1.

**Figure 2:**
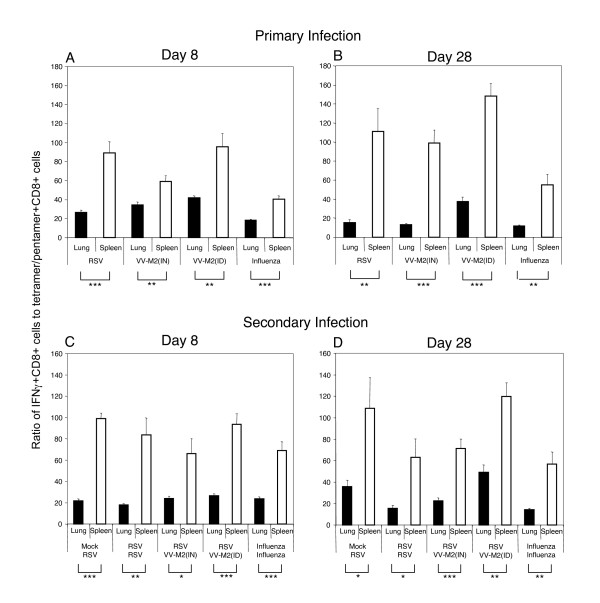
**CD8+ cells secreting IFNγ as % of tetramer/pentamer+CD8+ cells.** PMC or SMC were isolated from the lungs and the spleens, respectively, of mice on days 8 and 28 after the primary (A, B) or the secondary (C, D) infection, as indicated under the plots. The values were determined by dividing the numbers of IFNγ+CD8+ cells by the numbers of tetramer/pentamer+CD8+ cells and expressed as percentages. RSV and VV-M2-specific CD8+ T cells were analyzed using the RSV-specific tetramer, and the influenza virus-specific CD8+ T cells were analyzed using the influenza virus-specific pentamer. The data are from the experiment shown in Table 1. The values for the lungs and the spleens are shown by black and striped bars, respectively.

We also quantified the levels of IFNγ+CD8+ cells in PMC and SMC following in vitro stimulation with the epitope-specific peptides (Table 1). In each case, the percentage of IFNγ+CD8+ cells was several-fold lower than that of tetramer/pentamer+CD8+ cells. This difference also was observed when the number of tetramer/pentamer+CD8+ and IFNγ+CD8+ cells were calculated as a percentage of total PMC or SMC (as opposed to CD8+ cells, not shown). We also expressed the number of IFNγ+CD8+ cells as a percentage of the number of tetramer/pentamer+CD8+ cells (Figure 2). The resulting values confirmed the previous finding that tetramer+CD8+ CTL from the lungs of RSV-infected mice are impaired in IFNγ production [1,4,16,17]. Specifically, on day 8 after the primary infection, the number of pulmonary CD8+ cells capable of secreting IFNγ was only 26% the number of tetramer+CD8+ cells. In contrast, the number of splenic IFNγ+CD8+ cells was 89% that of the tetramer+CD8+ cells. Importantly, the virus-specific CD8+ cells isolated from the lungs of mice infected with VV-M2 by the IN route also showed an impairment in IFNγ production, as the number of IFNγ+CD8+ cells was only 34% that of tetramer+CD8+ cells, while in spleen the value was 59%. Moreover, in mice infected with VV-M2 by the ID route, the values were 42% and 96% in the lungs and spleens, respectively. Importantly, this reduced percentage of cells producing IFNγ was observed despite the lack of pulmonary replication of the virus in this group (above), indicating that the impairment in IFNγ secretion by pulmonary CD8 T cells is independent of local viral infection. A similar difference was observed in animals infected with influenza virus: the percentages of IFNγ-positive cells in the lungs on day 8 were much lower than in the spleens (19% versus 41%) (Figure 2A), a result that is consistent with a recently published study [4]. This difference was also observed on day 28 following a primary infection (Figure 2B), and on days 8 and 28 following a secondary infection (Figure 2C,D). This difference also was observed when the number of tetramer/pentamer+CD8+ and IFNγ+CD8+ cells were calculated as a percentage of total PMC or SMC (not shown).

These findings are consistent with a recent study demonstrating that, after a highly localized infection with VV by tail scarification, part of the activated virus-specific CD8+ CTL reach various lymph nodes throughout the body, which are free of the virus. These lymphocytes then acquire a phenotype specific for each homing tissue [9]. In the present study, virus-specific lymphocytes activated after a respiratory tract (RSV; influenza virus), local dermal (ID inoculation with VV-M2), or disseminated (IN inoculation with VV-M2) infection were present in the lungs and were impaired in secretion of IFNγ, irrespective of the type and site of infection. It is known that the pulmonary CTL induced by infections with respiratory viruses such as RSV and influenza virus can greatly augment pathology caused by these viruses in lungs [18-21]. It is possible that the tissue-specific functional impairment of the CD8+ CTL response in the lungs is a host-mediated mechanism for protection against an exaggerated and therefore harmful response. Possible mechanisms for this tissue-specific impairment could be a lack of factors necessary to maintain CTL effector functions in lung tissue [4], defective signaling [1,3], or excessive up-regulation and/or engagement of programmed death-1 receptor (PD-1) on the cell surface [22]. As the reported defect in pulmonary lymphocyte function was observed even in the absence of an active pulmonary infection (i.e. in mice infected with VV-M2 by ID route), we would expect that any differences in PD-1 expression between the lung and spleen would be present even in naïve mice. However, we did not observe a greater frequency of PD-1+ cells or the level of PD-1 expression on lymphocytes isolated from the lung, as compared to spleen, of uninfected mice (data not shown). While this result suggests that tissue-specific up-regulation of PD-1 on the surface of pulmonary lymphocytes is not the mechanism for pulmonary T cell dysfunction, this does not rule out the possibility of differences in PD-1 ligand expression between the lung and spleen, nor any of the other mechanisms mentioned above. Future studies will include further elucidation of the pathways responsible for the decrease in pulmonary CTL function. As the mucosal surfaces of the respiratory tract are a common site of entry and replication for various pathogens, the design of more effective vaccines and therapeutics will be greatly aided by gaining a better understanding of the local mechanisms of immunity.

## Conclusion

These data demonstrate, first, that the functional impairment of virus-specific CD8+ CTL in the lungs is not associated with a specific virus, since the effect was observed after infection with each of the three viruses used. This point is further validated by the observation that the same epitope expressed by two distinct viruses, RSV or VV-M2, manifested the same functional impairment in the lung versus spleen, even in the absence of viral replication in the lung. Thus, it is not the virus bearing the epitope nor local virus replication that results in the decreased functionality of CD8+ CTL in lungs, but rather the pulmonary site of residence of the cells. Therefore, the conclusion that RSV infection specifically impairs CD8+CTL functionality [1], and the hypothesis that this might contribute to RSV re-infection, must be reassessed. Second, essentially the same impairment was observed during primary and secondary (recall) responses for all the infections. Third, functional impairment of CD8+ CTL in lungs is not necessarily related to a respiratory tract infection, since it was also observed in lung CD8+ CTL that migrated from the site of a localized dermal infection with VV-M2. Fourth, the CD8+ CTL impairment observed in the study was a lung-specific phenomenon, as no impairment was observed in the spleen under conditions of local infection in the lung (i.e. influenza, RSV), localized dermal infection (i.e. VV-M2 administered ID), or disseminated infection (i.e. VV-M2 administered IN).

## Methods

### Viruses and mice

RSV strain A2 was propagated in HEp-2 cells with Opti-MEM medium (Invitrogen, Carlsbad, CA) containing 2% FBS. Virus titers were determined by titration in HEp-2 cells with immunostaining of plaques as previously described [23]. Influenza virus A/Puerto Rico/8/34 (H1N1) was propagated and titers determined in MDCK cells in the presence of 1 μg/ml of trypsin (Invitrogen). Recombinant WR strain of VV expressing RSV M2 protein (VV-M2) was constructed previously in our laboratory and was propagated and titered in Vero cells in the presence of 2% FBS. Seven- to 12-week-old BALB/c mice (Charles River Laboratories, Wilmington, MA) were used in all experiments.

### Infection of mice

Groups of mice were infected IN under light methoxyflurane anesthesia with RSV, influenza virus, or VV-M2 in a 100 μl inoculum. For ID infections, groups of mice received VV-M2 in a 50 μl inoculum.

### Vaccinia virus replication in mice

On the indicated days after infection, animals were sacrificed by carbon dioxide asphyxiation. The nasal turbinates and lung tissues were isolated and homogenized, and viruses were titrated in MDCK cell monolayers.

### Analysis of CTL response

Kinetics of the virus-specific CTL response have been determined in previous studies [24]. Mice were infected IN with RSV, influenza, VV-M2 or ID with VV-M2. On the indicated days, the animals were euthanized and total PMC or SMC were isolated from mouse lungs and spleens as previously described [14]. For quantitation of cells bearing T-cell receptors specific for the RSV M2-1 protein, PMC or SMC were stained with optimized amounts of phycoerythrin-conjugated MHC I H-2K^d ^tetramer complexes bearing the peptide epitope SYIGSINNI from the M2-1 protein (amino acids 82 to 90) [10,25] (provided by the NIAID Tetramer Facility, Yerkes Regional Primate Research Center, Atlanta, GA) and fluorescein isothiocyanate-conjugated rat anti-mouse CD8 monoclonal antibody, clone 53-6.7 (BD Biosciences). For analysis of influenza virus-specific CD8+ CTL, phycoerythrin-labeled MHC class I H-2K^d ^pentamers loaded with the NP peptide TYQRTRALV (amino acids 147–155) [13] (Proimmune, Oxford, UK) were used.

For quantitation of pulmonary CTL (from BALB/c mice) that secrete IFN-γ in response to stimulation specific for RSV or influenza virus, PMC were washed twice with phosphate-buffered saline containing 2% fetal bovine serum and resuspended in RPMI 1640 medium (Invitrogen, Carlsbad, CA) containing 10% fetal bovine serum, 100 U/ml of penicillin, 100 μg/ml of streptomycin sulfate and 20 mM of HEPES (Invitrogen) and incubated overnight with 1 μM of the SYIGSINNI (for RSV) or TYQRTRALV (for influenza virus) peptide in the presence of GolgiStop (BD Biosciences). Following stimulation, the PMC were washed twice, incubated with Fc Block (BD Biosciences) to block Fc receptors, stained with the fluorescein isothiocyanate-conjugated anti-mouse CD8 monoclonal antibody, fixed and permeabilized with Cytofix/Cytoperm (BD Biosciences), and stained with allophycocyanin-conjugated rat anti-mouse IFN-γ antibody, clone XMG1.2 (BD Biosciences). Flow cytometry analysis was performed using a FACSCalibur flow cytometer (BD Biosciences). A total of 30,000 cells were analyzed per sample.

## Competing interests

The authors declare that they have no competing interests.

## Authors' contributions

JMD carried out the experiments and wrote the manuscript. BRM and PLC provided advice and wrote the manuscript. AB conceived the study, carried out the experiments and wrote the manuscript. All authors approved the final version of the manuscript.
